# Versatile effects of galectin-1 protein-containing lipid bilayer coating for cardiovascular applications

**DOI:** 10.1016/j.bioactmat.2024.08.026

**Published:** 2024-09-03

**Authors:** Md Lemon Hasan, Ju Ro Lee, Khandoker Asiqur Rahaman, Dae Hyeok Yang, Yoon Ki Joung

**Affiliations:** a Center for Biomaterials, Biomedical Research Institute, Korea Institute of Science and Technology (KIST), Hwarangno 14-gil 5, Seongbuk-gu, Seoul, 02792, Republic of Korea; b Division of Bio-Medical Science & Technology, University of Science and Technology (UST), 113 Gwahangno, Yuseong-gu, Daejeon, 34113, Republic of Korea; c Center for Systems Biology, Massachusetts General Hospital Research Institute, Boston, MA, 02114, USA; d Department of Radiology, Massachusetts General Hospital, Harvard Medical School, Boston, MA, 02114, USA; e Institute of Cell and Tissue Engineering, College of Medicine, The Catholic University of Korea, Seoul, 06591, Republic of Korea; f KHU-KIST Department of Conversing Science and Technology, Graduate School, Kyung Hee University, Seoul, Republic of Korea

**Keywords:** Galectin-1 protein, Supported lipid bilayer, Macrophage, Cardiovascular devices, Inflammation

## Abstract

Modulating inflammatory cells in an implantation site leads to severe complications and still unsolved challenges for blood-contacting medical devices. Inspired by the role of galectin-1 (Gal-1) in selective functions on multiple cells and immunomodulatory processes, we prepared a biologically target-specific surface coated with the lipid bilayer containing Gal-1 (Gal-1-SLB) and investigate the proof of the biological effects. First, lipoamido-dPEG-acid was deposited on a gold-coated substrate to form a self-assembled monolayer and then conjugated dioleoylphosphatidylethanolamine (DOPE) onto that to produce a lower leaflet of the supported lipid bilayer (SLB) before fusing membrane-derived vesicles extracted from B16-F10 cells. The Gal-1-SLB showed the expected anti-fouling activity by revealing the resistance to protein adsorption and bacterial adhesion. *In vitro* studies showed that the Gal-1-SLB can promote endothelial function and inhibit smooth muscle cell proliferation. Moreover, Gal-1- SLB presents potential function for endothelial cell migration and angiogenic activities. *In vitro* macrophage culture studies showed that the Gal-1-SLB attenuated the LPS-induced inflammation and the production of macrophage-secreted inflammatory cytokines. Finally, the implanted Gal-1-SLB reduced the infiltration of immune cells at the tissue-implant interface and increased markers for M2 polarization and blood vessel formation *in vivo*. This straightforward surface coating with Gal-1 can be a useful strategy for modulating the vascular and immune cells around a blood-contacting device.

## Highlights

• A blood-contacting device covered with Gal −1 proteins containing lipid bilayer derived from B16-F10 cells was prepared.
• A self-assembled monolayer (SAM) was formed with thiol lipid to provide a spacer layer for the lipid bilayer.
• Coated substrates show anti-fouling effects in resisting proteins and bacterial cell adhesion.
• Gal-1 protein of the coated surface promotes endothelialization and macrophage polarization.
• Gal-1 protein positively controlled SMC activity and reduced host interface inflammatory activity revealed *in vivo.*

## Graphical abstract

We prepare a coating of the blood-contacting device surface with a lipid bilayer containing Gal-1 protein derived from B16-F18 cells. Tethered lipid bilayers formed on pre-made SAM-coated gold substrate. Besides the anti-fouling effect, the Gal-1 protein of the coated surface promotes endothelialization and macrophage polarization. In contrast, it positively controls smooth muscle cell activity and reduces host interface inflammatory activity.

**Figure:**
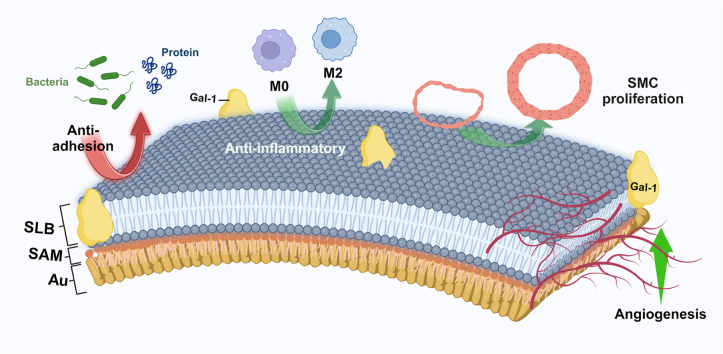

## 1 Introduction

Numerous implantable medical devices, such as stents, catheters, and heart valves, which come into direct contact with blood, face significant challenges related to thrombosis, known as forming blood clots and inflammation. These issues stem from difficulties achieving optimal blood compatibility, persistent inflammatory responses, and host-body rejection. These complications not only cause patient discomfort and localized tissue injury but can also lead to severe consequences like device failure, body rejection, and even patient death [[1], [2], [3]]. Targeting the endothelium, the thin layer of cells lining the interior surface of blood vessels is one effective way to overcome these obstacles. The endothelial layer plays a crucial role in maintaining vascular health and function. Damage to the endothelial layer can trigger thrombosis and inflammation as part of the host's response to implanted materials [4,5]. Thus, the blood-contacting materials have been coated or modified with various methods in order to promote blood compatibility and even vascular healing [[6], [7], [8]].

Researchers are currently focusing on developing multifunctional surfaces that not only reduce the risk of thrombosis but also encourage the growth of endothelial cells, a process known as endothelialization, restrict the proliferation of smooth muscle cells, which can lead to vessel blockage, and minimize inflammatory reactions [1,[9], [10], [11]]. To achieve these goals, scientists are exploring innovative approaches to co-immobilize two or more bioactive molecules on the surfaces of medical devices. These molecules include proteins, which can enhance cell adhesion and function; heparin, an anticoagulant that helps to prevent blood clots [6,12], and nitric oxide (NO) donors, which have anti-inflammatory and vasodilatory effects [[13], [14], [15]]. Each of these molecules offers different therapeutic benefits, and their combined use can create a synergistic effect on the performance of the medical device [9]. However, there are challenges with large-scale applications due to the expensive immobilization methods, short half-lives, and time-consuming processes. Despite these advancements, there are significant challenges to the large-scale application of these technologies. Immobilization methods can be expensive and complex, limiting their practicality for widespread use. Additionally, the bioactive molecules used in these coatings often have short half-lives, meaning they degrade or lose their effectiveness relatively quickly, reducing the modified surfaces' long-term benefits. The processes involved in creating these advanced coatings are also time-consuming, adding to production's overall cost and complexity.

Though the immobilization of bioactive molecules on the implant can offer an opportunity to create selective cell-surface interactions, they may undergo structural changes or even denaturation following adsorption, which results in functional damage, depending on how strongly they interact with the surface [[16], [17], [18]]. Supported lipid bilayers (SLBs) present opportunities for the controlled functionalization of solid surfaces and platforms for biomolecule integration in this context. However, integrating biomolecules, such as proteins and antibodies, into artificial membranes has limitations in the critical reconstruction process and their low ability to deliver overall effectiveness [17,19]. To overcome these restrictions, constructing SLBs with vesicles derived from cells could be ideal in the surface coating of biomaterials. Also, this biomembrane-imitating coating technology can employ various membrane proteins with available functions [20]. Furthermore, these also have a wide range of anti-fouling effects similar to a bilayer constructed with several phospholipids [20,21].

In this study, we explored the modification of gold surfaces with cell-derived supported lipid bilayers (SLBs) to enhance cell-surface interactions in biomedical implants. Gold is used in specific cardiovascular devices, but its application is relatively specialized. Gold has unique properties, such as excellent biocompatibility, resistance to corrosion, and electrical conductivity, which make it suitable for specific uses in medical implants and devices [22,23]. As endovascular stents are altered to add functionality, biocompatibility may suffer. Indeed, gold coatings of a stent type other than that used here elicited greater restenosis than similar uncoated stainless steel devices. There are some reports that gold-coated stents were associated with a considerable increase in the risk of restenosis over the first year after stenting [24]. Drawing inspiration from the roles of the galectin-1 protein (Gal-1) in anti-inflammatory responses [25] and cell-specific signaling processes [26], we developed a multifunctional surface coated with Gal-1 containing SLBs and evaluated its effectiveness both *in vitro* and *in vivo*. We utilized the murine melanoma cell line (B16-F10) as the source of membranes containing the Gal-1 protein.

Research has shown that Gal-1 stimulates endothelial cell function and promotes angiogenesis by regulating the H-Ras signaling cascade [27,28]. Additionally, Gal-1 plays a significant role in the attachment, spreading, and migration of smooth muscle cells (SMCs), mediated through interactions with extracellular matrix proteins [29]. Gal-1, a member of the β-galactoside-binding lectins family, regulates immune cells during injury-associated inflammation by reducing the synthesis of inflammatory cytokines and promoting vascularization through the vascular endothelial growth factor receptor (VEGFR)-2 signaling pathway [[30], [31], [32]]. Previous studies have identified that the primary causes of biomedical implant failure include abnormal proliferation and migration of SMCs, which can inhibit endothelialization, as well as the infiltration of immune cells at the implantation site and the production of soluble inflammatory cytokines [[33], [34], [35]]. Therefore, our current research aimed to assess the selective improvement of endothelial cell (EC) function and the polarization of macrophages to reduce inflammatory cytokine production *in vitro*. We also investigated the behaviors of immune cell infiltration *in vivo* using Gal-1-coated implant surfaces.

**Scheme 1:**
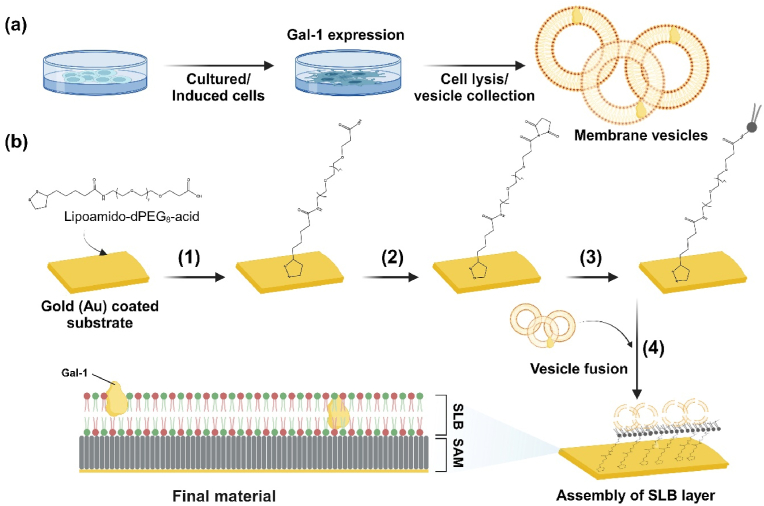
(a) The schematic process of cell membrane-derived liposome isolation, in which B16-F10 cells and HUVECs were cultured until a confluent state, and the cells were collected with lysis buffer, finally the membranes were extracted by ultra-centrifugation in a vesicle type. (b) The procedure for the SLB coating on a gold-coated substrate is as follows: 1. A gold-coated slide is immersed in 2 mM lipoamido-dPEG®_8_-acid solution for 24 h; 2. The carboxy-terminated SAM layer is modified by EDC-NHS treatment; 3. A lower leaflet of SLB was formed by immersion with 0.1 mg/ml DOPE solution; 4. Cell membrane-derived liposomes were disrupted and assembled to produce an SLB layer.

In this study, a self-assembled layer was formed with thiolated poly(ethylene glycol)s (PEGs) on a gold surface, and a lower leaflet was produced on the self-assembled PEG layer by depositing liposomes prepared with dioleoylphosphatidylethanolamine (DOPE). Finally, the Gal-1-containing membrane vesicles were fused with the lower leaflet to create the upper leaflet of the SLB (Scheme 1). The layer self-assembled on gold surface makes a spacing layer that provides a stable support for SLB. The Gal-1 containing SLB could play an anchoring role in selectively promoting adhesion, proliferation and migration of ECs, while controlling the function of SMCs *in vitro*. *In vitro* studies revealed that Gal-1 can attenuate LPS-induced inflammatory activation of macrophages and cytokine expression and promote M2 macrophage polarization. The host-implant response was examined by *in vivo* analysis, demonstrating that the Gal-1-modified implant could reduce immune cell-mediated inflammation and the M2 polarization of macrophages.

This study suggests a straightforward coating system for implantable and the purposeful use of Gal-1-containing membrane vesicles, and it establishes a new approach to using target-specific cell membrane proteins for the surface modification design of biomedical devices. B16-F10 melanoma cell line was used as a source of Gal-1, as it has low immunogenicity to the host cell [36]. In addition, we prepared two different SLB surfaces using human umbilical vein endothelial cell (HUVEC) from the HUVEC's membrane without Gal-1 and the induced HUVEC's membrane with Gal-1 [37]. The samples were named as Au (bare gold-coated slide), H-SLB (coated with membrane extracted from HUVECs), H-C-SLB (coated with membrane extracted from induced HUVECs with conditioned medium), and Gal-1-SLB (coated with membrane extracted from induced B16-F10 cells).

## 2 Materials and methods

### 2.1 Materials

The gold-coated slides were purchased from the Platypus Technologies. Lipoamido-dPEG®_8_-acid, (3-aminopropyl) triethoxysilane, succinic anhydride, 1-ethyl-3-(3-dimethylaminopropyl) carbodiimide (EDC), N-hydroxysulfosuccinimide sodium salt (sulfo-NHS, paraformaldehyde, Triton X-100 Sigma-Aldrich (St. Louis, USA). EDTA-free mini protease inhibitor tablet was purchased from Roche (Indianapolis, USA). 1,2-dioleoyl-sn-glycero-3-phosphoethanolamine-N-(lissamine rhodamine B sulfonyl) (Rhod-PE) was provided from Avanti Lipids (Alabaster, USA). CytoPainter Phalloidin-iFluor 488 reagent (ab176753), mouse monoclonal Anti-Galectin-1 antibody (ab108389), anti-iNOS antibody (ab283655), and anti-CD206 (ab64693) were purchased from Abcam (Cambridge, UK). Anti-mouse-Alexa Flour 488, anti-rabbit-Alexa Fluor 488, anti-mouse Alexa Flour 594, and live/dead assay kit were provided from Life Technologies, Inc. (Carlsbad, USA). The cell counting kit-8 (CCK-8) was obtained from Dojindo Laboratories (Kumamoto, Japan).

### 2.2 Isolation of cellular membranes

We used three types of cell membranes: HUVEC's membranes without Gal-1, Gal-1 induced HUVEC's membranes, and murine melanoma cell line's ones (B16-F10) with Gal-1. The cell membranes were extracted according to a previously reported protocol [38] with some modifications. Briefly, the cells were cultured up to 80% confluence. HUVECs and B16-F10 cells were cultured in endothelial cell basal medium-2 supplemented with EGM-2 SingleQuots and DMEM supplemented with 10% FBS and 1% antibiotic–antimycotic, respectively. For inducing HUVECs expressing Gal-1, cells were cultured until 70% confluency, and then the medium was replaced with a conditioned medium and incubated for 24 h. The conditioned medium was prepared with a 2:1 proportion of EGM-2 and medium collected from 24 h of B16-F10 culture. To harvest the membrane, cells were detached using a scraper and washed with a phosphate buffered saline (PBS) solution by centrifugation at 1100 rpm for 3 min. The pellet was suspended in a hypotonic lysing buffer, which was prepared by mixing 372.7 mg KCl (10 mM), 95.1 mg MgCl_2_ (2 mM) in 500 ml of 20 mM Tris-HCl at pH 7.5), finally, one EDTA-free mini protease inhibitor tablet per 10 ml of solution was mixed just before used. The cell suspension was disrupted using an ultrasonicator (Fisher Scientific) for 2 min, followed by centrifugation at 5000 rpm for 5 min, and the supernatant was saved. Next, the supernatant was centrifuged at 20,000 g for 20 min, and then the supernatant was collected again using an ultra-speed centrifuge (Beckman Coulter) at 100,000 g for 90 min. Finally, the pellet was collected and used for the subsequent experiment.

### 2.3 Formation of tethered lipid bilayers

Gold-coated glass slides were cut 0.5 cm^2^ of pieces. The gold substrates were cleaned with pure ethanol and immersed in lipoamido-dPEG®_8_-acid solution in ethanol (final concentration is 2 mM), backfilled the container with dry nitrogen, sealed the cap, and raped with paraffin, and left for 48 h at room temperature. Then, thiol-coated samples were sonicated for 2 min and washed with ethanol and nitrogen dry. Then, the surface reacted overnight with EDC (20 mg/ml) and NHS (10 mg/ml) in MES buffer at pH 5.5. The NHS-modified samples were incubated in 0.1 mg/ml DOPE in chloroform at 4 °C overnight. All steps were carried out under a nitrogen atmosphere. The surface modification was analyzed using Fourier transform infrared (FT-IR) spectroscopy. Finally, the activated substrate was immersed in the prepared vesicles, diluted with calcium-containing Tris-HCl buffer (10 mM Tris-HCl, 150 mM NaCl, 2 mM Ca^2+^), and kept for 30 min at room temperature. The formed SLBs were gently washed to remove unassembled excess vesicles. The formation of TLBs was observed and visualized with a Zeiss LSM 700 laser-scanning confocal microscope (Carl Zeiss Microimaging GmbH, Jena, Germany). Rhod-PE (1 wt%) was used for the microscopic fluorescence observation and recovery after the photo-bleaching (FRAP) experiment.

### 2.4 Anti-fouling studies

#### 2.4.1 Protein adsorption test

The protein adsorption was monitored for each substrate by quartz crystal microbalance with dissipation (QCM-D, Q-Sense, Biolin Scientific) apparatus using a gold (Q-5MHz Au, Runlux Crystal) coated sensor. A solution of 0.5 mg/ml human fibrinogen and 1 mg/ml bovine serum albumin (BSA) in PBS was run at a flow rate of 50 μl/min, and the temperature was controlled at 25 °C for all experiments. The adsorption of proteins was calculated with the QSense Dfined software adopted with the sauerbrey equation, *ΔF = −C*_*f*_ × *Δm*, where *ΔF* is the time-resolved changes in resonance frequency (*Hz*), *C*_*f*_ is the sensitivity factor of the crystal, and *Δm* is the mass difference per unit area (μg/cm^2^).

A solution of 0.5 mg/ml FITC-fibrinogen and 1 mg/ml FITC-bovine serum albumin (BSA) in PBS was prepared for fluorescence images. Then, the prepared samples were incubated in the solution at 37 °C for 1 h. After washing with PBS three times, samples were observed using a confocal microscope.

#### 2.4.2 Bacterial adhesion test

The ability of the coated substrates to resist bacterial adhesion was assessed using Escherichia coli (E. Coli, O157: H7). The bacteria were cultivated in Luria broth (L.B., Broth Miller, NJ) overnight until the optical density (OD) at 600 nm reached 0.8, and then the OD was adjusted to 0.1 by dilution. The substrate samples were placed in 24-well plates and treated with 2 ml of the bacterial solution (OD_600_ = 0.1) for 24 h at 37 °C for the colony-forming unit (CFU) assay. Following incubation, non-adherent bacteria were gently removed by washing the samples in PBS. The washed samples were then vortexed for 5 min in a conical tube containing 2 mL of sterile PBS. Following serial dilution, 100 μl of the diluted bacteria solution was plated on TSB agar plates. Colonies were counted to determine the CFUs of bacteria on samples after 24-h incubation period.

### 2.5 *In vitro* cell studies

#### 2.5.1 Endothelial cell study

To study EC's responses, HUVECs were cultured in endothelial cell basal medium-2 supplemented with EGM™-2 SingleQuots® at 37 °C in a humidified atmosphere containing 5% CO_2_ and 95+% air. When cells reached 80% confluence, they were detached with 0.5% trypsin-EDTA. In addition, human coronary artery smooth muscle cells (HCASMCs) were cultured as mentioned before, but in SMC basal medium and supplemented with SmGM®-2 SingleQuots®.

To investigate adhesion and spreading behavior, four types of substrate mentioned above were placed in 48-well plates, and cells were seeded at a density of 5000 cells/well and incubated at 37 °C for 2 h. Cells were washed with a pre-warmed PBS solution and fixed with 4% paraformaldehyde for 15 min. For permeabilization, cells were incubated in 0.1% Triton X-100 for 10 min, washed with PBS solution, and blocked with 3% BSA solution for 1 h. For the visualization of cytoskeletons, cells were incubated with CytoPainter Phalloidin-iFluor 488 (ab176753) for 1 h and counter-stained with DAPI. Fluorescence images were taken using a Zeiss LSM 700 laser-scanning confocal microscope.

For the cell proliferation, cells were seeded at a density of 1 × 10^4^ cells/well in 48 well plates and incubated for 1, 3 and 7 days. As previously mentioned, a confocal microscope was used to analyze the filamentous actin of the cells to evaluate the morphological features after a specific time of incubation fixation. In addition, using the Image J software, cell number and spreading area were quantified from five random images of triplicate samples (10 × ).

#### 2.5.2 Co-culture of HUVECs and HCASMCs

HUVECs were labeled with PKH26 red fluorescence, while HCASMCs were labeled with PKH67 green fluorescence according to product instruction. Both cell suspensions were mixed at a volume ratio of 1:1 and added to the samples at a 5 × 10^4^ cells/cm^2^ density. The cells were observed and photographed after co-culturing for 24 h in a cell incubator at 37 °C under 5% CO_2_. The Image J software was used to quantify the competitive growth ratio of HUVECs to HCASMCs. Three brightfield images and five confocal images were randomly chosen for the statistical analysis of the ratio of HUVECs to HCASMCs.

#### 2.5.3 Assessment of angiogenic responses

Inter-surface cell migration assays were performed by directly contacting the cell to the coated surface. Cells were seeded on a culture plate and allowed to grow to a monolayer. Then, the surface contact with the cells was placed, allowing it to migrate from the culture well to the sample's surface for 24 h. The total migrated area was observed with fluorescence staining and quantified from five random images of triplicate samples (10 × ) by the Image J software.

The tube formation assay followed a previously published protocol [39] with our modification. HUVECs were cultured on different surfaces for 5 days. Then, the cells were harvested and seeded in a pre-coated slide chamber with membrane matrix (Geltrex™) at a density of 2 × 10^5^ cells per chamber. After 24-h incubation, tubular networks were captured by a bright field microscope. The tube formation parameter was analyzed with the Image J software [40].

Further, HUVECs were cultured for 2 days to assess angiogenic and anti-angiogenic marker expression. After fixation cells were labeled with primary antibody of ICAM-1 (#MA5407; ThermoFisher)), VEGFR2 (ab9530), or vWF (ab6994), followed by goat anti-mouse IgG H&L (Alexa Fluor® 488) or anti-rabbiot (Alexa Fluor® 594), and the nuclear was counter-stained with DAPI. Cells were observed with a laser-scanning confocal microscope (Zeiss LSM 700).

The relative expression of angiogenic and anti-angiogenic markers responsible for the angiogenesis pathway was quantified using real-time PCR. The primers are listed in Table S1. The GAPDH gene was used as a control to normalize relative mRNA values and calculated using the comparative cycle threshold (*△△Ct*) method.

#### 2.5.4 *In vitro* macrophage response

Murine macrophage cell line RAW 264.7 (ATCC TIB- 71TM) was cultured in DMEM medium (Corning, NY, USA) supplemented with 10-% fetal bovine serum (FBS) and 1% penicillin/streptomycin, in a humidified incubator (37 °C, 5-% CO_2_). When the confluence reached ∼80%, the cells were trypsinized for cell seeding on the substrate surface at 1 × 10^4^ cells/well density. Cells were incubated in a growth medium for 48 h with or without LPS 1 μg/L. To visualize the morphology and iNOS, CD206 expression, cells were fixed with 4% paraformaldehyde, permeabilized with 0.1% TritonX-100 and labeled anti-iNOS antibody (ab283655) or anti-CD206 primary antibody, followed by goat anti-rabbit IgG H&L (Alexa Fluor® 488). TRITC-phalloidin was used for F-actin staining, and the nuclear was counter-stained with DAPI. Cells were observed with a laser-scanning confocal microscope (Zeiss LSM 700).

The relative expression of inflammatory markers (iNOS, IL-1β and TNF-α) and pro-inflammatory markers (CD206, Arg1) were quantified by the real-time PCR. RAW 264.7 cells were cultured in the same condition mentioned above. The primers are listed in Table S1. The GAPDH gene was used as a control to normalize relative mRNA values and calculated using the comparative cycle threshold (*△△Ct*) method.

### 2.6 *In vivo* subcutaneous implantation

An implantation model using C57BL/6J female mice, center Lab. Animal Inc. (South Korea), 8 weeks old, was used, following proper housing and treatment procedures following the institutional guidelines (KIST). Briefly, a 0.5 cm^2^ piece of bare Au, H-SLB and Gal-1-SLB samples were implanted into a subcutaneous pocket by incision on the back. Then, the incision was closed with PCL sutures, and povidone was applied. After a certain period (7 and 14 days), mice were euthanized under general anesthesia, and samples were taken out before their skin and muscle tissue complexes were extracted. For histological analysis, extracted specimens were fixed for 72 h in paraformaldehyde and preserved at −80 °C in PBS for PCR analysis.

Hematoxylin and Eosin (H&E) staining was used for the histological observation. First, fixed tissues were embedded in paraffin and cross-sectioned 5 μm. Then, the tissue sections were stained using the H&E staining protocol developed by IHCWORLD. Briefly, paraffin-embedded tissue sections were deparaffinized at 60 °C and hydrated sequentially in a series of xylene, alcohol and water. Then, the nuclei were stained with Harris hematoxylin and differentiated with 1% acid alcohol. Finally, tissue sections were counter-stained in eosin Y solution, followed by dehydration and mounting with a xylene-based mounting medium.

The harvested tissues stored at −80 °C were used for the relative expression of inflammatory and pro-inflammatory markers. According to the manufacturer's instructions, total RNA was extracted from the homogenized tissue sample using a TRIZOL extraction kit (Intron Biotechnology, Seoul, Korea). The primer sets are listed in Table S1. The GAPDH gene was used as a control to normalize relative mRNA values and calculated using the comparative cycle threshold (*△△Ct*) method.

### 2.7 Statistical analysis

The data were expressed as mean ± standard deviation (S.D.). Statistical comparisons were determined by the one-way ANOVA using the GraphPad Prism software. At least triplicate samples were used for statical analysis, otherwise mentioned. The statistical significance was defined at the value of *p* < 0.05.

## 3 Results and discussion

### 3.1 Surface characterization

**Fig. 1:**
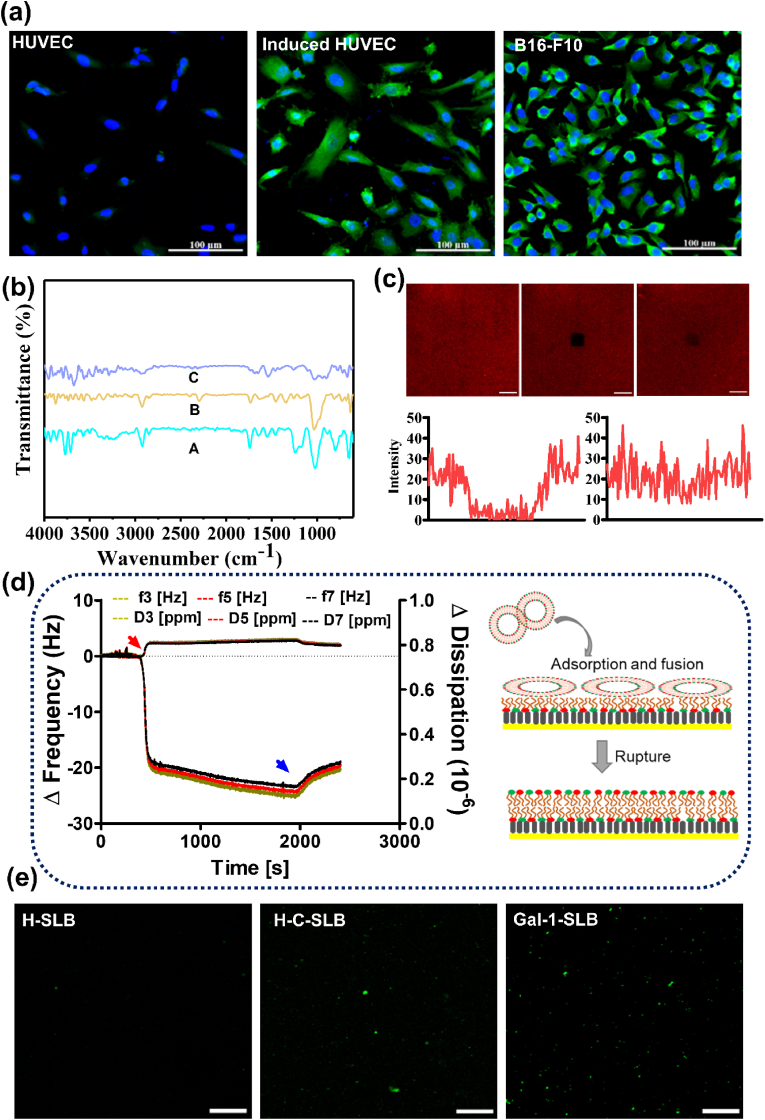
Surface characterizations. (a) Fluorescence staining of Gal-1 expressed cells including HUVECs, induced HUVECs and B16-F10. (b) ATR FT-IR spectra of modified surfaces (A: bare gold substrate, B: lipoamido-dPEG®_8_-acid assembled on gold, C: DOPE-conjugated lipoamido-dPEG®_8_-acid on gold). (c) Results of fluorescence recovery after photo-bleaching (FRAP) confirming the SLB formation of cellular membrane vesicles, showing confocal images and the intensity at the bleaching point, scale bar is 10 μm. (d) QCM-D monitoring of SLB formation on pre-SAM coated Au sensor, Data represent frequency and dissipation from multiple overtones (*n* = 3–7), as the time of the vesicle fusion of cell-derived membranes. The red and blue arrows represent the vesicle injection and washing steps. (e) Fluorescence images of Gal-1 staining for three differently modified surfaces, scale bar is 50 μm.

The self-assembled layer on gold was characterized by ATR-FTIR spectroscopy. The major characteristic spectra of lipoamido-dPEG®_8_-acid thiol lipid, observed in solution, identically appeared in the spectra of the surface (Fig. S1). Fig. 1b shows the characteristic signals of −CH stretch at 2975−2830 cm^−1^ for aldehyde, and C=O at 1727 cm^−1^ for carboxyl group appeared after the formation of the assembled layer. The self-assembled layer treated on a gold-coated sensor was measured by QCM-D (Figs. S2a and S2b). The frequency change was observed at −7.5 H_Z_ and thickness was calculated as 1.48 ± 0.1 nm, depicting spacer carbons of the spacer lipids [41]. In order to produce a lower leaflet of the SLB layer, the assembled layer was further conjugated with DOPE via the EDC/NHS chemistry. As a result, two N−H bending appeared at 1540 cm^−1^ and 1645 cm^−1^ for secondary amines. In addition, signals of C−H bending at 1340 cm^−1^, C−O stretching at 1235 cm^−1^ and 1030 cm^−1^ are attributed to the carbonyl group [42].

### 3.2 Surface characterizations

Along with the bare gold, we prepared three modified substrates to evaluate the potential effect of Gal-1. The expression of Gal-1 was confirmed by fluorescent staining of cultured cells with an anti-Gal-1 antibody (Fig. 1a). As we expected, HUVECs rarely expressed Gal-1, while the higher expression of Gal-1 and morphological changes were observed in the induced HUVECs. B16-F10 cells showed prominent expression of Gal-1. DLS determined the extracted membrane vesicle sizes and zeta potential before fusion (Fig. S3). There are no significant differences in surface charge and size among the vesicles. The membrane fusion and SLB formation were analyzed with FRAP assay (Fig. 1c). The fluorescence images of Rhod-PE, fluorescently labeled phospholipid conjugated with vesicle, show the assembled layer formed on the surface. Additionally, a photo-bleached spot's fluorescent image and intensity were almost recovered within 10 min, indicating that the formed layer is a laterally movable bilayer [20].

Therefore, the SLB formation was further examined by monitoring with a quartz crystal microbalance with dissipation (QCM-D) (Fig. 1d). The monolayer of membrane vesicles was formed by first-order kinetics on the SAM immobilized with DOPEs before forming a hybrid bilayer. The interaction between DOPE as a fusogenic helper and membrane vesicle is sufficient for immediate rupture (schematic in Fig. 1d) [20,43]. The plateau values of frequency shift below *Δf* = −11 ± 1 compared to homogeneous SLB, indicating membrane vesicles formed a monolayer on top of the DOPE. The topographic image (Fig. S4) confirmed the almost homogeneous SLB surface formation with the lipid film thickness of *h* = 2.7 ± 0.2 nm. Our observation of membrane vesicle rupture and SLB formation on a coated SAM layer aligned with topographic and mechanical observations in previous studies [44].

Moreover, M.E. Villanueva et al. demonstrated that the nanoroughness of the substrate of more than 3 nm affects the formation of SLBs and needs extra fusion energy for vesicle rupture to SLBs formation [45]. Therefore, we confirmed the surface roughness of the gold-coated slide bought from Platopus. The AFM observation showed that the substrate surface is very flat with a surface roughness of 0.028 nm (root mean square, RMS) (Fig. S5), while the surface roughness of the Au-coated sensor for QCM-D is less than 1 nm (RMS), provided by Biolin Scientific. In addition, the SAM coating thickness was measured at 1.48 ± 0.1 nm. Overall, the minimum influence of surface roughness for rupturing vesicles in our coating substrate was maintained to ensure the SLB formation.

The retention of Gal-1 after bilayer formation was further confirmed by immunostaining with the anti-Gal-1 antibody. Fig. 1e shows the presence of Gal-1 in supported membranes. The H-SLB surface has no expression of Gal-1. On the other hand, the Gal-1 expression observed on H-C-SLB and Gal-1-SLB surfaces as puncta has different distribution and positioning of proteins, assuming different fluorescence intensities [46].

### 3.3 Anti-fouling effects

**Fig. 2:**
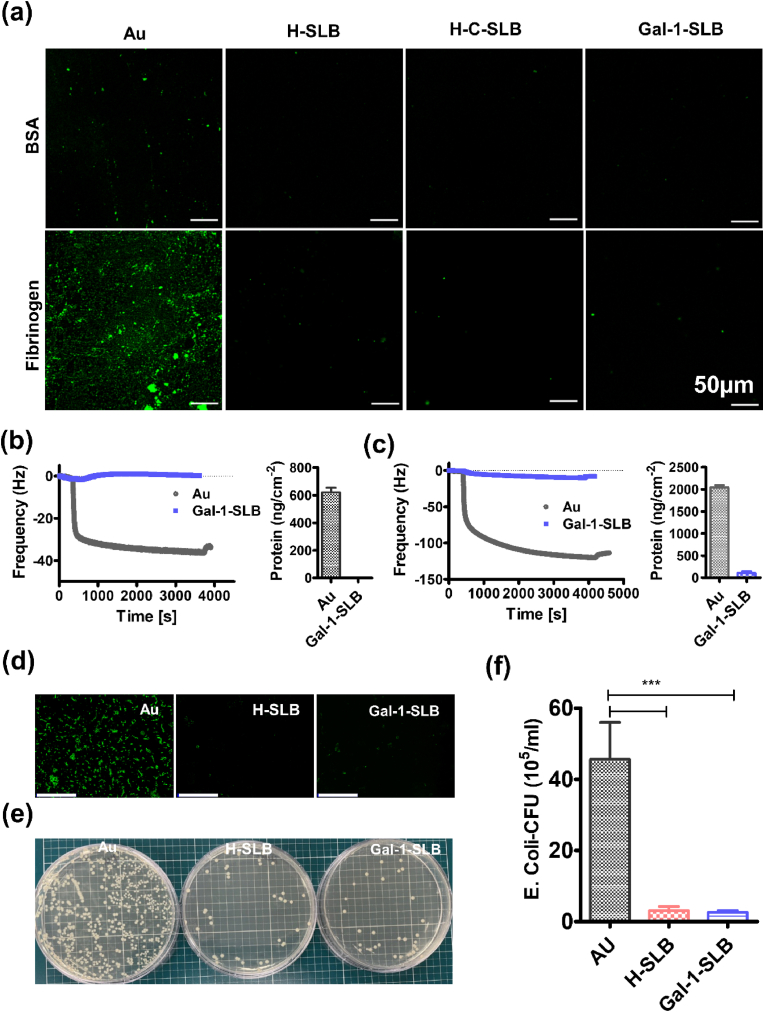
Anti-fouling effects. (a) Fluorescence images showing the adsorption of fluorescence-labeled BSA and human fibrinogen, scale bare is 50 μm. Changes in frequency associated with the protein adsorption, and quantitative measurement of absorbed protein by QCM-D on various surfaces by surbery equation of (b) albumin and (c) fibrinogen. (d) Fluorescence staining images of bacterial adhesion, scale bare is 200 μm. (e) Plate photos and (f) CFU of E. Coli grown on the modified substrates for 24 h.

When a medical device come in contact with blood, this initiates the protein adsorption process followed by thrombus formation. The protein adsorption plays a critical role in subsequent cascade reactions on the device surfaces [47,48]. Thus, we investigated the protein-resistant properties of the modified surfaces with the immunofluorescence and the quartz crystal microbalance with dissipation (QCM-D) using bovine serum albumin (BSA) and fibrinogen that are major plasma proteins responsible for inducing adhesion and activation of platelets and inflammatory events [1,15,21]. The adsorption was qualitatively visualized by incubating fluorescence-labeled proteins on different surfaces. As shown in Fig. 2a, the adsorption of both proteins was hardly seen on the membrane-coated surface, in contrast to the bare Au surface. However, the absorption of human fibrinogen is more prominent than that of BSA on the bare Au surface.

In addition, the protein adsorption on the different surfaces was analyzed as real-time monitoring. In the QCM-D analysis, the frequency responses to the adsorption of proteins on the bare and coated Au sensor are shown in Fig. 2b & c. The adsorbed proteins can be accurately measured by monitoring frequency (Δ*f)* and dissipation (Δ*D*) changes versus time. Changes in adsorbed protein mass are approximated by (*f*), and the viscoelastic characteristics are matched by (*D*). On the bare Au sensor, the addition of protein induced negatively changed frequencies of nearly up to −25 Hz (BSA) and −100 Hz (fibrinogen), indicating adsorption of protein masses. The lack of dissipation changes was observed, which means the rigidity of the adsorbed protein films. At the rinsing step, changes in frequency and dissipation were converted to reverse, meaning that the physisorbed proteins were rinsed away while the coated sensor produced almost no frequency changes. In addition, the density of adsorbed proteins was quantitatively determined by the sauerbrey equation. The amount of albumin and fibrinogen in the bare Au sensors were 626.5 ± 58.3 ng/cm^2^ and 2003.63 ± 15.1 ng/cm^2,^ respectively, while the Gal-1-SLB coated sensor had undetectable or Negligible amounts of protein adsorption. All the quantitative data was measured from the 3rd overtone, as we observed deviation multiple overtone (n = 3–11) (Figs. S6 and S7), which fulfills the requirements for applying the Sauerbrey equation. The result demonstrated the superior protein-inhibiting activity of modified surfaces. SLB-based substrates generally have the anti-fouling effect [11], and our modified surface also shows the same effectiveness against all tested biological components.

Further, we evaluated the bacterial adhesion of the coated substrates. The substrates were incubated in E. Coli suspension and plated for the colony formation unit (CFU) analysis. Strategies for preventing bacterial infection are an urgent prerequisite for designing implanted medical devices [49]. Fig. 2d shows the prominent fluorescence in the bare gold surface, whereas significantly reduced fluorescences were observed for SLB-modified substrates. Moreover, bacterial growth in the TSB plate was significantly reduced (Fig. 2e). The CFU was 47 ± 14.7 × 10^5^ in bare Au, 6.33 ± 3.6 × 10^5^ in H-SLB, and 5.73 ± 3.2 × 10^5^ in Gal-1-SLB coated surface, respectively (Fig. 2f).

Overall, This observation of anti-fouling effect is coincident with our previous studies in which SLBs from cell membranes resist protein adsorption and bacterial adhesion [20]. Additionally, the spacer produced between the gold surface and the SLB layer with thiol lipid does not affect the anti-fouling properties of the SLBs layer. Moreover, the spacer provides stable support for the SLBs layer [42].

### 3.4 *In vitro* responses to HUVECs

**Fig. 3:**
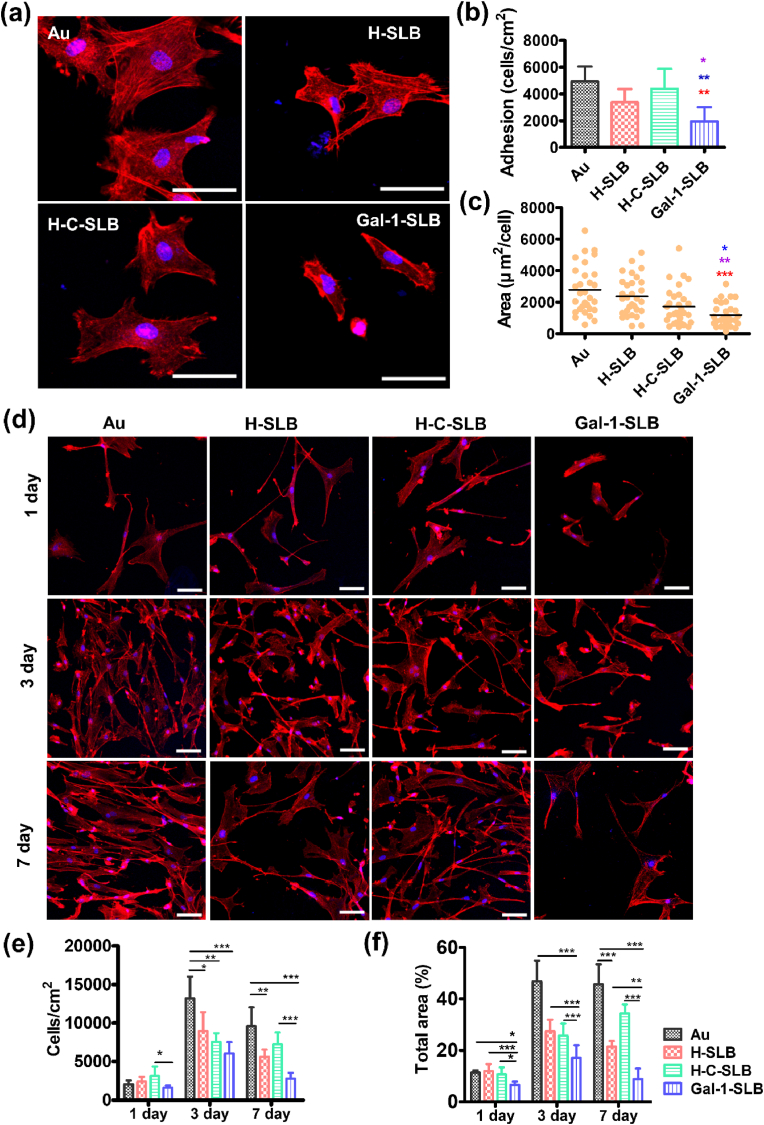
The response of HUVECs to Gal-1 coated substrates. (a) Fluorescence micrographs of HUVECs adhesion at 2 h, showing the morphology, scale bar is 50 μm. (b) The number of adherent cells and (c) cell area per cell in 2 h were calculated using the Image J software. Red (*), brown (*), and blue (*) asterisks indicate significant compared to Au, H-SLB, and H-C-SLB, respectively, (n = 5). (d) HUVECs were cultured for 7 days, showing the cell morphology and proliferation, scale bar is 50 μm. (e) Quantitative analysis of HUVECs proliferation and (f) total surface area were counted after culturing cells for 7 days. Data are presented as the mean ± standard deviation (S.D.). Student's t-test was performed between two groups, and ANOVA was performed to compare the differences in a different group (n = 5, *P < 0.05, **P < 0.01, ***P < 0.001 were considered statistically significant).

The vascular endothelium play a significant role in maintaining homeostasis at the host interface. Endothelial cell injury during implantation is a significant concern as it can lead to a cascade of adverse events that affect the overall success and longevity of the implantation. The recovery of damaged endothelial cells after device implantation is crucial to preserve blood fluidity and the non-thrombogenic inner layer of the vascular wall [50]. Thus, providing a favorable surface for endothelial cells is on topic for medical devices. Here, we investigated the responses of HUVECs to the SLBs coated surfaces, a widely used model in vascular biology and endothelial cell research. These cells provide an excellent system for studying various aspects of endothelial function, angiogenesis, and vascular diseases and closely mimic human endothelial cells *in vivo* [51]. Fig. 3a shows the cell adhesion with regular morphology where all three surfaces showed significantly increased cell adhesion for 2 h compared to bare Au substrate (Fig. 3b). The cell adhesion between the coating surface is insignificant. However, the spreading area per cell is significantly increased in the Gal-1-SLB surface compared to other surfaces (Fig. 3c).

The proliferation behavior of HUVECs was assessed for 7 days. Fig. 3d shows the morphological behavior in the proliferation period. Cell numbers slightly increased in the coated surfaces compared to the bare Au on 1-day, but the cell spreading was significantly increased in the Gal-1-SLB surface compared to the Au and H-SLB surface (Fig. 3e & f). On 3-day, all coating surfaces showed significantly increased cell numbers and speeding areas compared to bare Au surfaces. However, the Gal-1-SLB also showed significant surface coverage compared to the H-SLB. On 7-day, the H-SLB showed a distorted cell spreading trend and slowed cell growth like bare surface. In contrast, Gal-1-SLB shows increased cell growth and spreading. Overall, the response of HUVECs to Gal-1-SLB was superior among surfaces in adhesion and proliferation, displaying more comprehensive cytoplasmic extension. The surface marker from Gal-1-SLB, specifically Gal-1, presumably promotes protein H-Ras signaling to activate some cascade reaction that stimulates endothelial cell proliferation and migration [27]. However, HUVECs only express Gal-1 in the membrane in induced conditions, while the Gal-1 presented on H-C-SLB shows inconsistent behavior in HUVECs. It could be explained that Gal-1 has different activities depending on the microenvironment, as the H-C-SLB contains Gal-1 from an inflammatory-induced condition [30,52,53].

### 3.5 *In vitro* responses to HASMCs

Many studies have demonstrated that Gal-1 could modulate VSMCs [29], specifically, Tsai MS et al. reported that the deficiency of Gal-1 increases SMC cell adhesion and proliferation [26]. Here, we evaluated HCASMCs' behavior on the coated surface, which is valuable in cardiovascular research, providing significant insights into the mechanisms underlying vascular diseases and closely mimicking human smooth muscle cells *in vivo* [54,55].

**Fig. 4:**
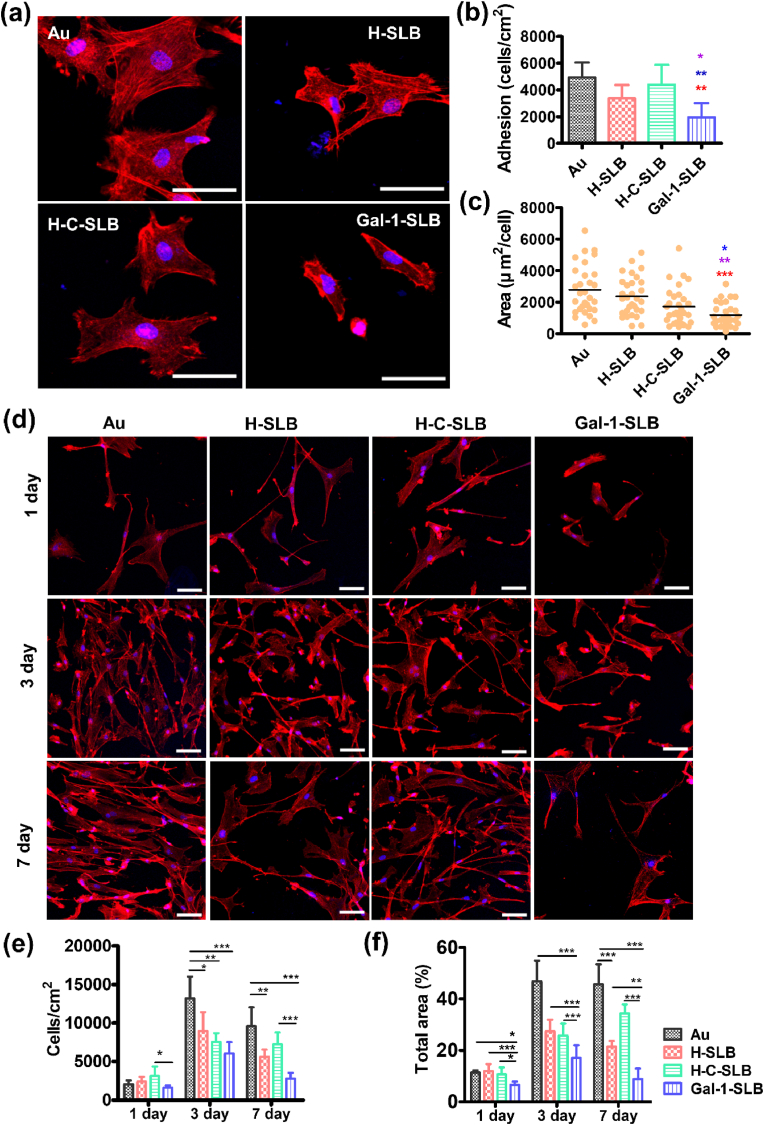
HACSMC responses on the modified surfaces. (a) Fluorescence micrographs of HACSMCs adhesion at 2 h, showing the morphology, scale bar is 50 μm. (b) The number of adherent cells and (c) area per cell at 2 h were calculated by the Image J software (n = 5, Red (*), brown (*), and blue (*) asterisks indicate significance compared to Au, H-SLB, and H-C-SLB, respectively). (d) Cells were cultured for 7 days, showing the cell morphology and proliferation, scale bar is 50 μm. Quantitative analysis of HACSMCs proliferation (e) and total surface area (f) counted after cultivating for 7 days. Data are presented as the mean ± standard deviation (S.D.). Student's t-test was performed between two groups, and ANOVA was performed to compare the differences in a different group (n = 5, *P < 0.05, **P < 0.01, ***P < 0.001 were considered statistically significant).

HASMCs adhesion and spreading on Gal-1-SLB were severely impaired. As shown in Fig. 4a, the HASMCs had spreader morphology on all other surfaces than Gal-1-SLB. At 2 h, the HASMCs adhesion was significantly decreased on Gal-1-SLB, compared to other surfaces. However, H-SLB and H-C-SLB surfaces show slightly higher cell adhesion and spreading than bare Au (Fig. 4b and c). In contrast, cell morphology was smaller, with less spreading in Gal-1-SLB compared to all surfaces by 2 h. During the 7-day proliferation period, HASMCs have similar but a little different morphology on each surface (Fig. 4d). In Day 1, the number of cells was lowest on the Gal-1-SLB than others (Fig. 4e). Meanwhile, the total area of cells was significantly reduced on Gal-1-SLB compared to all surfaces (Fig. 4f). In Day 3, Gal-1-SLB showed significantly reduced cell number and speeding area compared to all surfaces. In Day 7, cells showed slower growth on all surfaces. However, the cell numbers on Gal-1-SLB were significantly lower compared to Au and H-C-SLB, but they were non-significant on the surface of H-SLB. The result demonstrates that Gal-1 proteins in the Gal-1-SLB surface could potentially lower the adhesion, growth, and spreading of HACSMCs.

### 3.6 Co-culture of HUVECs and HASMCs

**Fig. 5:**
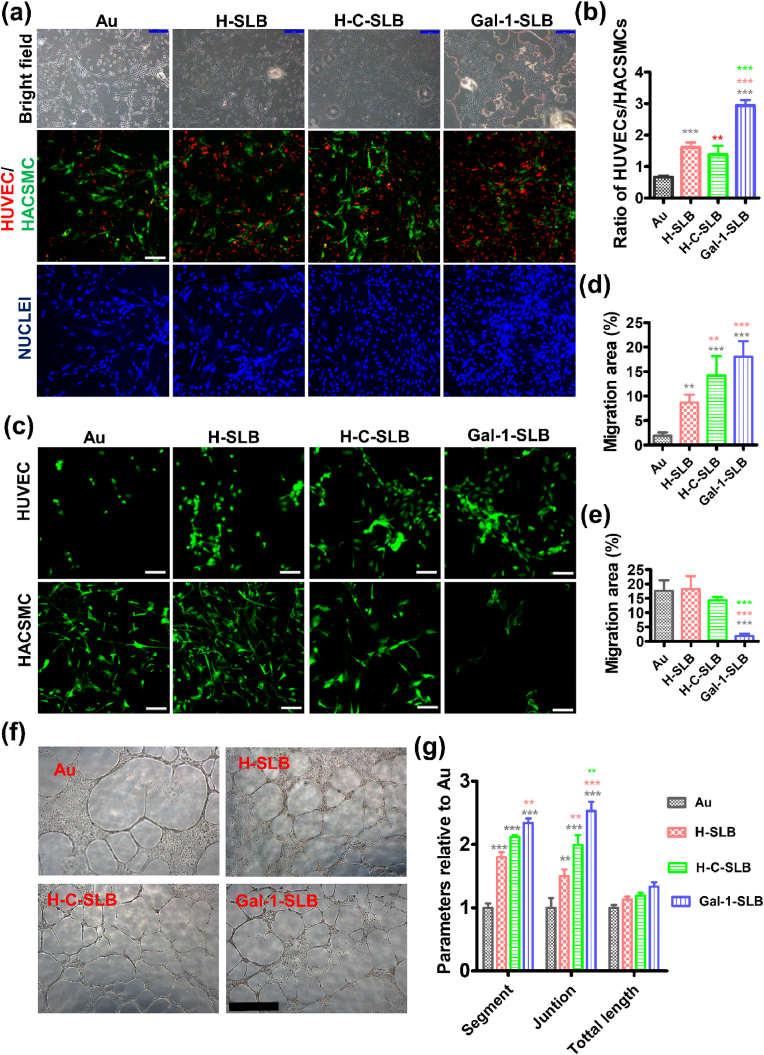
HUVECs and HUASMCs cells were co-cultured to investigate the competitive adhesion and proliferation behaviors, (a) Fluorescence staining of HUVECS using PKH26 (red) and HASMCs using PKH67 (green), bright field images (scale bar is 200 μm) stained images (scale bar is 100 μm). (b) The Image J software was used to calculate the ratio of HUVECs/HACSMCs (n = 5, black (*), red (*), and green (*) asterisks are indicated significant compared to Au, H-SLB, and H-C-SLB, respectively); *In vitro* angiogenic effects of SLB-modified surfaces. (c) Confocal images showing the migration of HUVECs and HACSMCs after placing the coating surface on the confluent cell layer for 24 h. (d and e) Quantification results of the area of migrated HUVECs and HACSMCs. (f) Tubular network formation of HUVECs, extracted from different surfaces on membrane matrix, scale bar is 1 mm. (g) Parameters of tube formation were analyzed by the Image J plugin software with angiogenesis analyser, n = 5, black (*), red (*), and green (*) asterisks are indicated significant compared to Au, H-SLB, and H-C-SLB, respectively.

Many studies suggested that the synergistic growth of ECs and SMCs is the primary prerequisite for forming an endothelium *in vivo*. The behavior of the growth pattern in coexistence of both cells under *in vitro* culture could be attributed to the surface characteristics of substrates [56]. So, we evaluated cell behaviors after co-culturing HUVECs and HUASMCs at the ratio of 1:1 for 24 h (Fig. 5a). The ratio of HUVECs to HUASMCs in the bare Au surface was 0.66 ± 0.04, significantly lower than that in the coated samples (Fig. 5b). However, the ratio of HUVECs to HUASMCs in the Gal-1-SLB was 2.94 ± 0.16, significantly higher than all substrates. The potential role of Gal-1 in resisting SMCs from over-activation could support HUVECs in forming an endothelial layer. Dominant adhesion and growth of ECs on the implant surface are vital for recovering the injured endothelial layer. Meanwhile, in habiting SMCs, adhesion and overactivation are necessary for protecting neointimal hyperplasia. Thus, *in vitro* culturing of ECs and SMCs demonstrated that Gal-1 on the coated surface could provide target-specific advantages to the surrounding microenvironment, which is desperately needed for developing a successful cardiovascular implant.

### 3.7 Angiogenic effects

We evaluated the angiogenic effects of the coated surfaces by the migration and tube formation assay. For the migration assay, both HUVECs and HASMCs were cultured in a culture plate and allowed to migrate to the coated surfaces with direct contact of the cells to the surfaces. HUVECs and HASMCs showed different patterns of migration on the Gal-1-SLB (Fig. 5c). The total area of the migrated HUVECs was significantly broader on Gal-1-SLB and H-C-SLB than on bare Au and H-SLB (Fig. 5d). On the other hand, the migration of HASMCs was markedly lower in Gal-1-SLB than in all other surfaces (Fig. 5e).

The HUVECs were seeded on the matrix after culturing on the coated surfaces, and tube formation was prominent on the coated surfaces (Fig. 5f). HUVECs were cultured on different modified surfaces, and the efficacy of forming vascular-like tubes on a pro-angiogenic substrate was observed. The cells harvested from the coated surfaces showed a better network of vascular-like structures, showing significantly increased segment and junction numbers relative to the bare Au surface, though the total length is not significant (Fig. 5g). Moreover, Gal-1-SLB showed prominent tube formation compared to all other surfaces. Thus, the demonstrated result attributed that Gal-1 in the Gal-1-SLB promoted angiogenesis. Endothelial cells lining newly formed blood vessels transport nutrients and oxygen to tissues, support immunological surveillance by hematopoietic cells, and eliminate waste. We plated HUVECs on a basement membrane matrix. When these cells encounter angiogenic signals in the medium, they form capillary-like structures. Endothelial cells begin to align within an hour, and lumen-containing tubules start to emerge within 2 h, indicating rapid tube formation. A balance of pro- and anti-angiogenic signals tightly regulates the process of angiogenesis. These include integrins, chemokines, angiopoietins, oxygen-sensing agents, junctional molecules, and endogenous inhibitors [57].

**Fig. 6:**
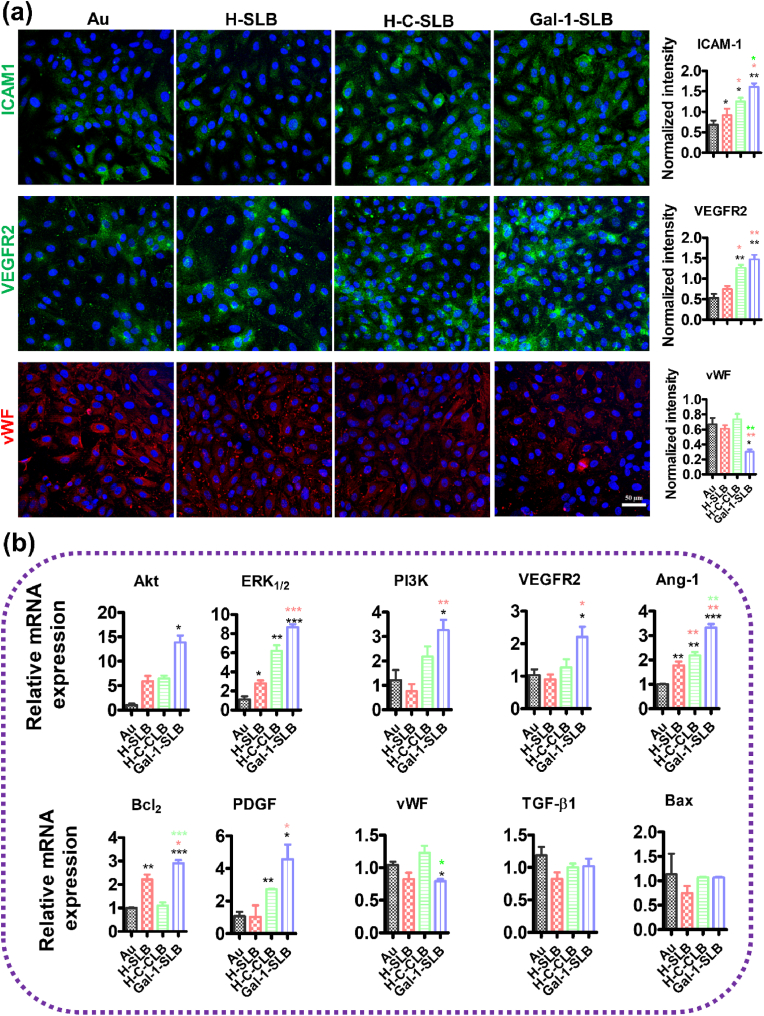
*In vitro* angiogenic effects of SLB-modified surfaces. (a) Confocal images showing the HUVECs cultured and the expression of different markers responsible for angiogenesis (ICAM1, VEGFR2) and anti-angiogenesis (vWF). (b) Relative expression levels of angiogenesis (ICAM1, VEGFR2) and anti-angiogenesis markers regulated different angiogenesis pathways. Black (*), red (*), and green (*) asterisks indicate significance compared to Au, H-SLB, and H-C-SLB, respectively. Data are presented as the mean ± SD n = 3, *P < 0.05, **P < 0.01, and ***P < 0.001.

Several studies reported that Gal-1 modulates signaling pathways related to migration and anagenesis [25,26,58]. For example, Gal-1 treatment on endothelial cells promotes H-Ras signaling, stimulating endothelial cell proliferation and migration [27]. From the previous report, Gal-1 directly regulates the angiogenesis pathway, so we analyzed the angiogenic signaling molecule. Fig. 6a shows the expression pattern of pro-angiogenic markers ICAM1 and VEGFR2 and anti-angiogenic marker vWF. ICAM1 and VEGFR2 expressions markedly increased in the SLB-coated surface compared to Au, while the Gal-1-containing surface showed prominent expressions between the SLBs surface. On the other hand, vWF, responsible for prohibiting angiogenesis [59], was significantly downregulated in Gal-1-SLB compared to other surfaces. From the qRT PCR analysis of the angiogenesis pathway (Fig. 6b), we found the relative mRNA expression of all of the pro-angiogenic markers was significantly increased in Gal-1-SLB surfaces compared to Au, while other SLB surfaces showed an inconsistent trend. However, the anti-angiogenesis expression of TGF-B1 and BAX were non-significant, but vWF was significantly downregulated in the Gal-1-SLB surface compared to all surfaces. The overall result demonstrated that the Gal-1 protein could potentially regulate the angiogenesis pathway from the Gal-1-SLB surface.

### 3.8 *In vitro* responses to macrophages

Various endotoxins can quickly contaminate the surface of biomaterials and cardiovascular devices, resulting in infection. Consequently, the contaminated surface can trigger blood components involved in the inflammatory and thrombotic response [1]. Moreover, the material surface experiences inflammatory soluble cytokines released from the host cells due to injury to the implantation site. Long-term contact with these chemicals leads to chronic inflammation, which prompts the creation of a fibrous encapsulation surrounding the implantation material, ultimately causing failure of endothelialization [13,60]. Thus, biomaterial-human interfaces must be formulated to reduce inflammation and effectively to be capable of macrophage polarization.

**Fig. 7:**
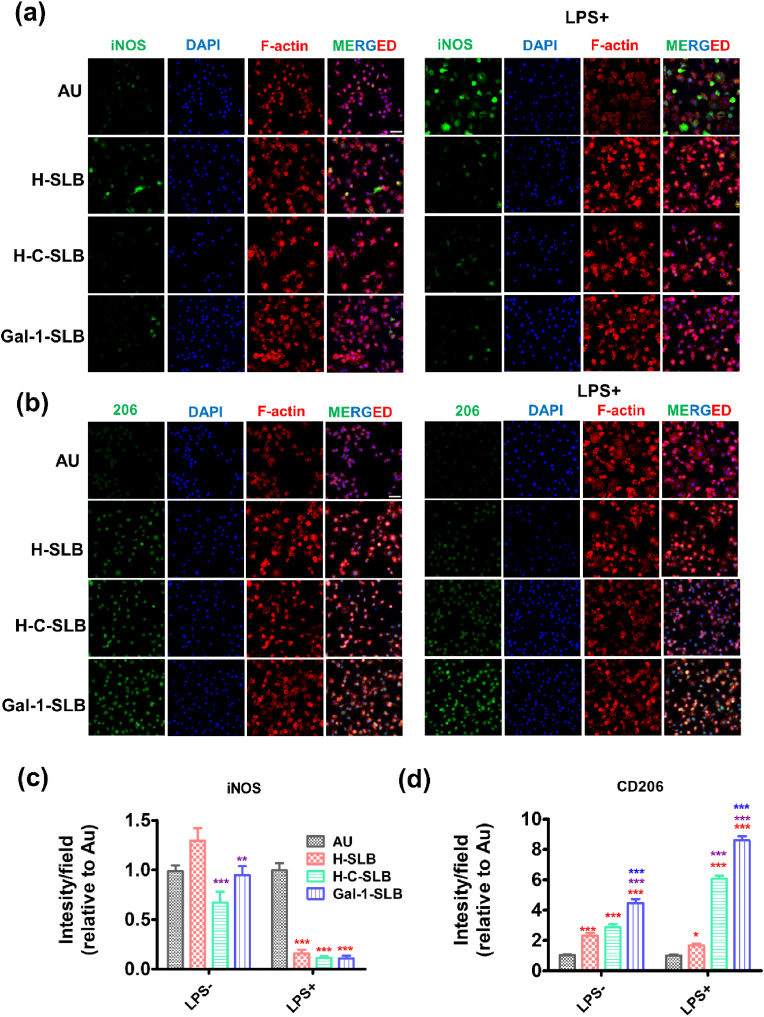
Anti**-**inflammatory activity of the coated surfaces. Raw 264.7 cells were cultured on different substrates for 24 h to investigate macrophage cell behaviors. The cells were stained with iNOS (M1, green) marker (a) and 206 (M2, green) marker (b); nuclei were stained with DAPI (blue), and cytoskeletons stained with TRITC-phalloidin (red), the fluorescence intensity of iNOS (c) and CD206 (d) was measured from 5 different fields using the Image J software. Red (*), brown (*), and blue (*) asterisks indicate significance compared to Au, H-SLB, and H-C-SLB, respectively. Data are presented as the mean ± standard deviation (S.D.) (n = 5, *P < 0.05, **P < 0.01, ***P < 0.001).

The inflammatory response of the coated surface was evaluated using RAW264.7 cells. After seeding, the cells were also treated with lipopolysaccharides (LPS) from Gram-negative bacteria, usually called endotoxin, which stimulates macrophage cells to release soluble cytokines. However, there was no significant cytotoxic effect on the modified surface for RAW264.7 (Fig. S8). We cultured RAW264.7 cells on different surfaces with/without LPS for 48 h to assess their anti-inflammatory activity. Overall, the fluorescence staining of iNOS (a marker for M1 phenotype) and CD206 (a marker for M2 phenotype) demonstrated a significant draw out to inflammatory responses (Fig. 7a and ab). The iNOS expression was not noticeable on all surfaces in a standard medium. When LPS was applied, the iNOS expression was significantly induced in the bare Au surface; in contrast, the coated surface showed no influence of LPS.

**Fig. 8:**
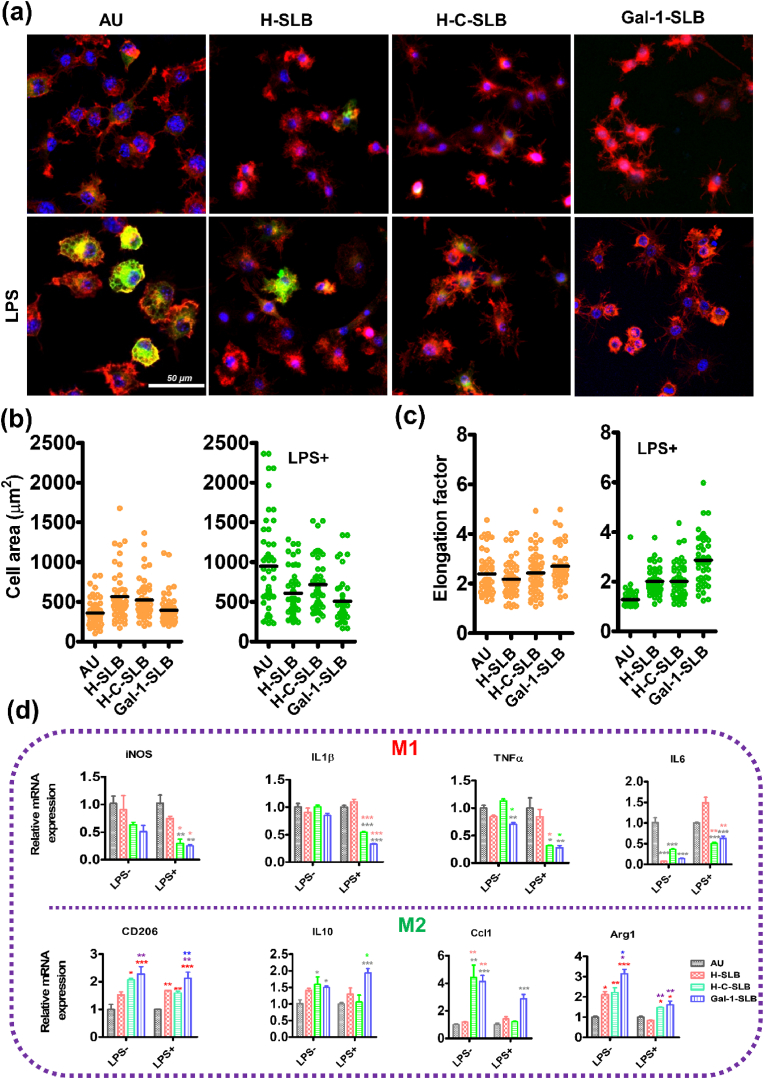
(a) Morphological changes of Raw 264.7 cells were observed by confocal microscopy. Quantification of cell area (b) and elongation (c) as the length of the long axis divided by the length of the short axis. At least 50 cells from 5 different fields were analyzed using the Image J software for the measuring. (d) Relative expression levels of M1 and M2 markers in macrophages grown on modified substrates with and without LPS-induced medium. Black (*), red (*), and green (*) asterisks indicate significance compared to Au, H-SLB, and H-C-SLB, respectively. Data are presented as the mean ± SD n = 3, *P < 0.05, **P < 0.01, and ***P < 0.001.

Similarly, the CD206 polarized marker was also expressed in the coated surface without bothering LPS induction. On the other hand, the bare Au surface rarely showed polarization in both conditions (Fig. 7c and d). We observed the morphological change (Fig. 8a) and analyzed cell area and elongation factor (Fig. 8b and c). Macrophage cells were induced by LPS, which stimulates M1 inflammatory marker expression, causing cells to have a round, pancake-like shape. In contrast, the coated surfaces were unresponsive to LPS induction, which led to cellular elongation, indicating M2 polarization [61].

We also determined the relative mRNA expression level of inflammatory (M1) and pro-inflammatory (M2) markers (Fig. 8d). The result demonstrated no significant change in the expression level of iNOS and IL1β in a standard medium among the surface. In contrast, TNF-α and IL6 expression levels were significantly lower in Gal-1-SLB compared to bare Au. On the other hand, in the LPS-induced medium, all M1 markers (iNOS, IL-1β, TNF-α, and IL6) had a higher expression on Au and H-SLB and significantly lower in H-C-SLB and Gal-1-SLB, indicating that the surface containing Gal-1 attenuated LPS induced inflammatory mediator expression. Moreover, the expression of polarized markers (Arg1, IL10, Ccl1, and CD206) was significantly higher in Gal-1-SLB than bare Au and H-SLB in both non-induced and LPS-induced medium. Thus, the coated surface effectively suppressed pro-inflammatory mediator expression in LPS-induced RAW264.7 cells, suggesting that Gal-1 could prevent the inflammatory activity of macrophages. Therefore, the functional activity of Gal-1 from the SLB surface positively responds against inflammation. As previously reported, the immune-modulatory effect of Gal-1 is by down-regulating inflammatory-mediated cytokine production and controlling macrophage functions [53].

### 3.9 *In vivo* studies on biocompatibility and inflammatory responses

**Fig. 9:**
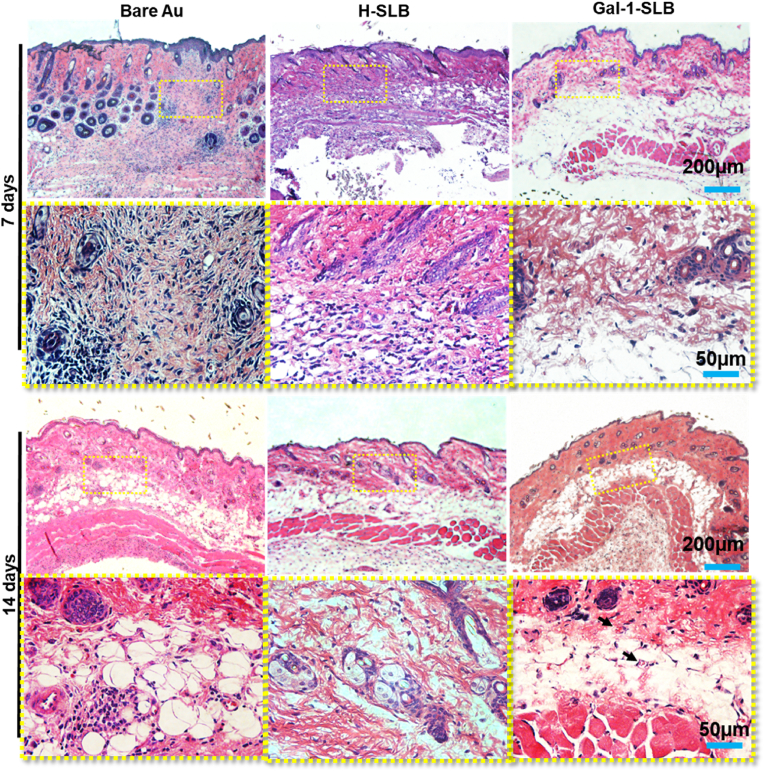
Representative image of H&E staining for the histological evaluation of implantation in S.D. rats**.** Newly formed blood vessels are marked with the black arrow.

A subcutaneous implantation model in mouse was used to evaluate biocompatibility and early-stage host tissue response to the implanted surfaces. The coated surfaces with HUVEC and B16-F10 membranes were implanted into the back facing up, while bare Au was facing up the gold-coated side of the slide. Implanted samples harvested at 7 and 14 days were healed, with back hair grown normally and no infection observed in the skin (Fig. S9). In addition, the harvested tissue samples at 7 and 14 days were evaluated for inflammatory activity using histology, and cytokine marker expression levels were examined. The histological image shows the healing process in bare Au-implanted mice with prominent infiltration of immune cells at 7 days, which was also observed in resolving processes at 14 days (Fig. 9). The inflammatory reaction in mouse implanted with H-SLB samples also presented severe inflammatory cell infiltration; however, the immune cell presence at 14 days was markedly reduced, and new blood vessels were observed. On the other hand, the mice implanted with Gal-1-SLB samples show a low immune reaction and immune cell infiltration in the surgical site in both the 7 and 14 days of post-implantation. Moreover, new blood vessels could be seen prominently from 7 days in the case of Gal-1-SLB.

**Fig. 10:**
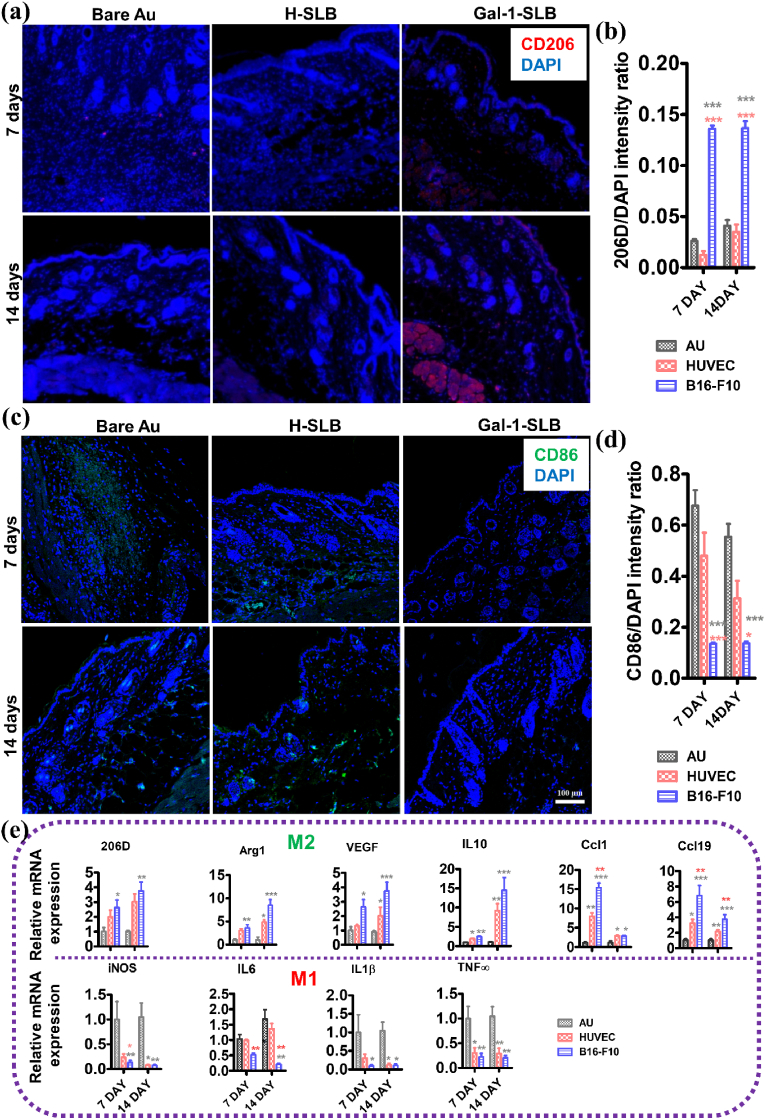
(a) Representative immunohistochemical images of CD206 (red) immunolabeling at a single tissue section from implanted mice for 7 and 14 days; nuclei were stained with DAPI (blue). (b) The intensity ratio of CD206/DAPI from immunolabeled tissue sections at 7 and 14 days, n = 3, black (*), and red (*) streaks indicated as significant compared to bare Au and H-SLB, respectively (C) Representative immunohistochemical images of CD86 (green) immunolabeling at a single tissue section from implanted mice for 7 and 14 days; nuclei were stained with DAPI (blue). (d)The intensity ratio of CD86/DAPI from immunolabeled tissue sections at 7 and 14 days, n = 3, black (*), and red (*) streaks indicated as significant compared to bare Au and H-SLB, respectively (e) Relative mRNA expression levels of M2 markers (CD206, Arg-1, VEGF, IL10, Ccl1 and Ccl19) and M1 markers (iNOS, IL6, IL-1β and TNF-α) in the tissues extracted at 7 and 14 days of implantation. (n = 3 for each group).

Immunohistochemistry was performed to assess the M2 polarization marker CD206 and M1 marker CD86 expression in the implantation site (Fig. 10). The CD206 labeling showed a significant increase in Gal-1-SLB implanted animals at 7 and 14 days, compared to bare Au and H-SLB implanted animals (Fig. 10a and b). However, the cell infiltration in bare Au and H-SLB was reduced at the implantation of 14 days. Similarly, the CD86 positive cells were significantly invaded in Au surface at 7 days, which may decrease in 14 days. Gal-1-SLB implanted animals showed reduced considerably infiltration of CD86-positive cells. The qRT-PCR was performed to analyze the regulatory efficacy of Gal-1 on cytokine expression level related to inflammation and macrophage polarization from 7 to 14-day post-implantation samples (Fig. 10e). The gene expression of inducible nitric oxide synthase (iNOS), interleukin-1 beta (IL-1β), IL6 and TNFα were significantly lower in the Gal-1-SLB implanted mice than bare Au at 7 and 14 days. The iNOS and IL1β expression was markedly lower in H-SLB implanted mice than bare Au at 14 days, while iNOS expression was significantly higher in H-SLB than Gal-1-SLB in 7 days, which is resolved at 14 days. Another inflammatory marker expression, TNF-α, was significantly lower in H-SLB and Gal-1-SLB than bare Au samples at 7 and 14 days. The polarization marker CD206, Arg-1, VEGF, IL10, Ccl1, and Ccl19 expression were significantly higher in the Gal-1-SLB than bare Au at 7 and 14 days, while the H-SLB showed inconsistent expression. Overall, the *in vivo* result demonstrates that the Gal-1 in the coated surface effectively attenuates the inflammatory reaction between the host and the implanted surface. Moreover, enhanced polarized macrophage recruitment and the expression of angiogenic markers play a crucial role in new blood vessel formation and facilitate re-endothelialization [62].

## 4 Conclusions

We optimized the coating method on the gold surface with Gal-1 containing cell membrane-derived liposomes. The modified surface showed the expected anti-fouling activity by resisting the adhesion of proteins and bacteria. Notably, Gal-1-SLB shows a ability to play a cell-specific role in stimulating endothelial cells and smooth muscle cell proliferation. In addition, Gal-1 from the Gal-1-SLB promotes HUVECs adhesion, proliferation, and migration behaviors with prominent angiogenic effects. Moreover, the Gal-1-SLB could attenuate the induced inflammation in response to host-surface interaction. Gal-1-SLB also demonstrates that Gal-1 controls macrophage activity by promoting macrophage polarization to M2 and reducing M1 markers in induced *in vitro* and *in vivo* conditions. Finally, these results depicted our hypothesis that the Gal-1 containing modified surface would promote endothelial cell behaviors and reduce the inflammatory reaction in the host implant interface. However, the B16-F10 cell membrane contains many types of proteins, including those of the GAL family. Though we found no superior effect of other proteins over Gal-1, in future research, critical analysis of protein array from membrane surface would be an identical and potential tool for biologically controlled biomedical device surface coating.

## Ethics approval and consent to participate

The animal study was performed according to Korea Institute of Science and Technology Animal Care and Use Committee Guidelines. The study was also approved by the Korea Institute of Science and Technology Animal Care and Use Committee (Approval number: KIST-5088-2022-09-124).

## CRediT authorship contribution statement

**Md Lemon Hasan:** Writing – review & editing, Writing – original draft, Visualization, Validation, Methodology, Investigation, Formal analysis, Conceptualization. **Ju Ro Lee:** Validation, Formal analysis, Data curation. **Khandoker Asiqur Rahaman:** Data curation, Investigation, Validation. **Dae Hyeok Yang:** Validation, Supervision, Methodology, Investigation, Data curation. **Yoon Ki Joung:** Writing – review & editing, Visualization, Validation, Supervision, Software, Resources, Project administration, Methodology, Investigation, Funding acquisition, Formal analysis, Data curation.

## Declaration of competing interest

The authors declare the following financial interests/personal relationships which may be considered as potential competing interests:

Yoon Ki Joung reports financial support supported by Nano Material Technology Development Program (NRF-2021M3H4A1A04092879) through the 10.13039/501100003725National Research Foundation of Korea (10.13039/100028114NRF) funded by the 10.13039/501100014188Ministry of Science and ICT and the Meterials and Parts 10.13039/100006180Technology Development Program (20023353) funded by the Ministry of Trade, Industry & Energy (10.13039/501100003052MOTIE, Korea).
