# Supplementary material for: Versatile effects of galectin-1 protein-containing lipid bilayer coating for cardiovascular applications
Source: Bioact Mater. 2024 Sep 3;42:207–25. doi: 10.1016/j.bioactmat.2024.08.026 (PMC11403261; doi:10.1016/j.bioactmat.2024.08.026)
Supplement: Multimedia component 1 [file mmc1.docx]

Versatile effects of galectin-1 protein-containing lipid bilayer coating for cardiovascular applications

Md. Lemon Hasan^a,b^, Ju Ro Lee^a,c,d^, Khandoker Asiqur Rahaman^a^, Dae Hyeok Yang^e,*^ , Yoon Ki Joung^a,b,f,**^

*^a^ Center for Biomaterials, Biomedical Research Institute, Korea Institute of Science and Technology (KIST), Hwarangno 14-gil 5, Seongbuk-gu, Seoul 02792, Republic of Korea*

*^b^ Division of Bio-Medical Science & Technology, University of Science and Technology (UST), 113 Gwahangno, Yuseong-gu, Daejeon 34113, Republic of Korea*

*^c^ Center for Systems Biology, Massachusetts General Hospital Research Institute, Boston, MA, 02114, USA*

*^d^ Department of Radiology, Massachusetts General Hospital, Harvard Medical School, Boston, MA, 02114, USA*

*^e^ Institute of Cell and Tissue Engineering, College of Medicine, The Catholic University of Korea, Seoul 06591, Republic of Korea*

*^f^ KHU-KIST Department of Conversing Science and Technology, Graduate School, Kyung Hee University, Seoul, Republic of Korea*

E-mail: yangdh@catholic.ac.kr (D.H. Yang), ykjoung@kist.re.kr (Y.K. Joung).

Fig. S1. FT-IR spectra of thiol solution (lipoamido-dPEG^®^_8_-acid).


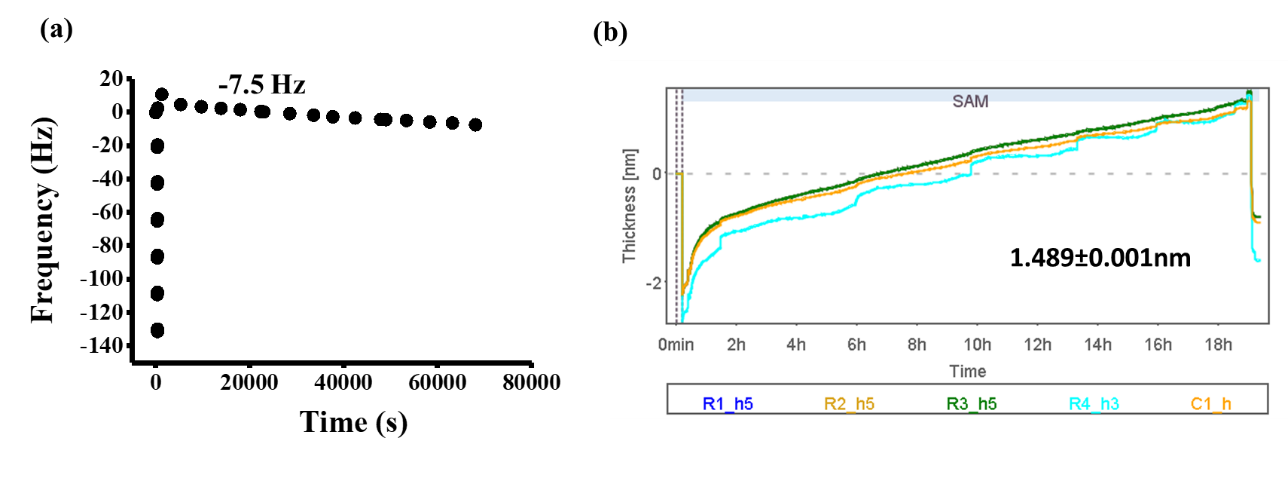


Fig. S2. Real-time frequency shift (a) and thickness (b) measured by QCMD during SAM formation.

Fig. S3. Zeta size and zeta potential of membrane vesicles extracted from different cells measured by DLS; water is the dispersion medium.


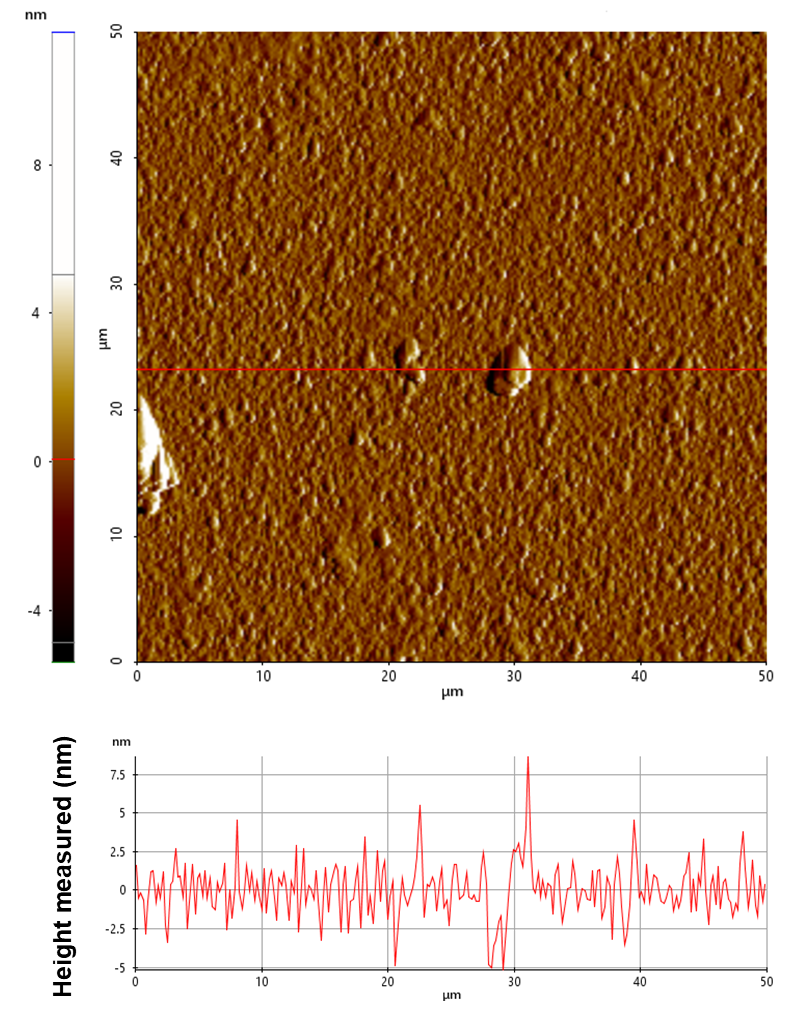


Fig. S4. Topographical images of SLBs formed on top of the SAM layer. The height measured from cross-section is indicated by red line.


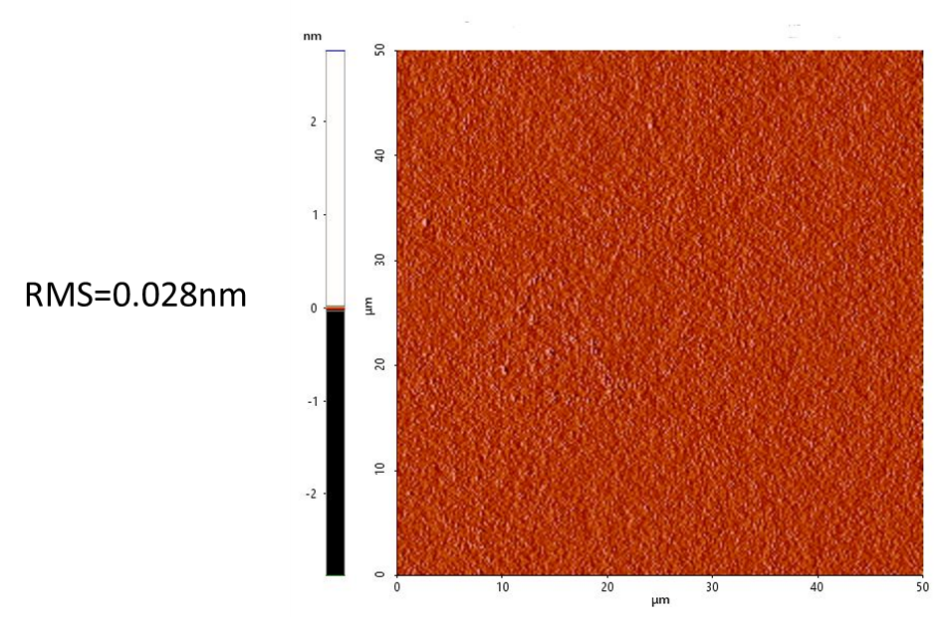


**Fig. S5.** Topographical images showing the surface roughness (RMS=0.028nm) of gold-coated slide bought from Platopus.


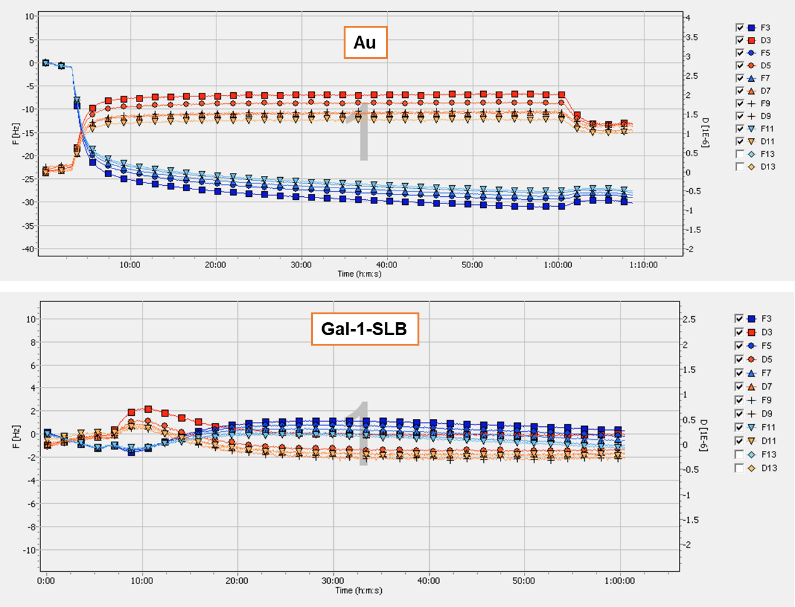


Figure.S6. QCM-D monitoring frequency (Δ*f)* and dissipation (Δ*D*) of albumin adsorption on Au and Gal-1-SLB surfaces, presenting raw data of all running overtone.


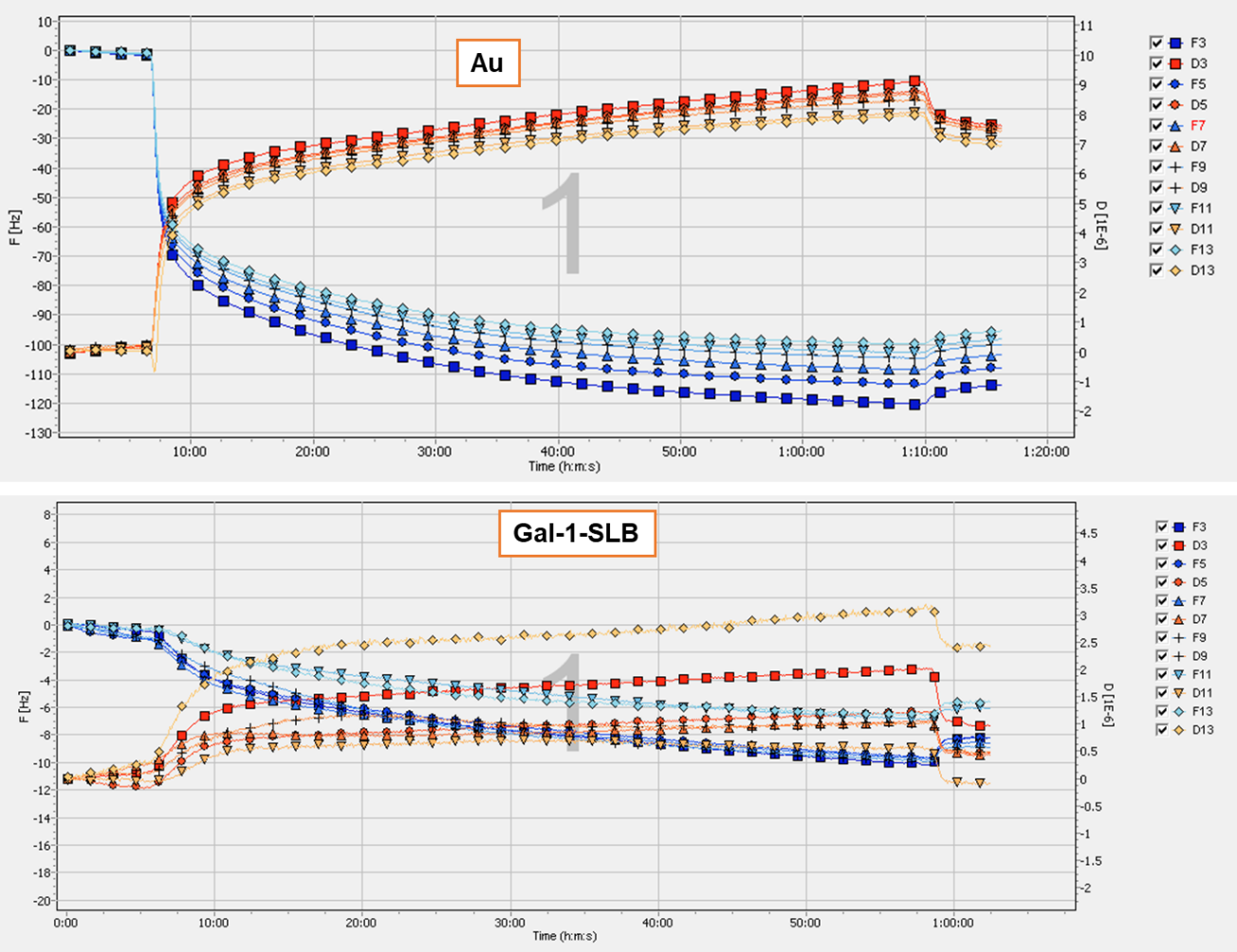


Figure.S7. QCM-D monitoring frequency (Δ*f)* and dissipation (Δ*D*) of fibrinogen adsorption on Au and Gal-1-SLB surfaces, presenting raw data of all running overtone.

Fig. S8. Cell viability of RAW 264.7 cells on different surfaces.


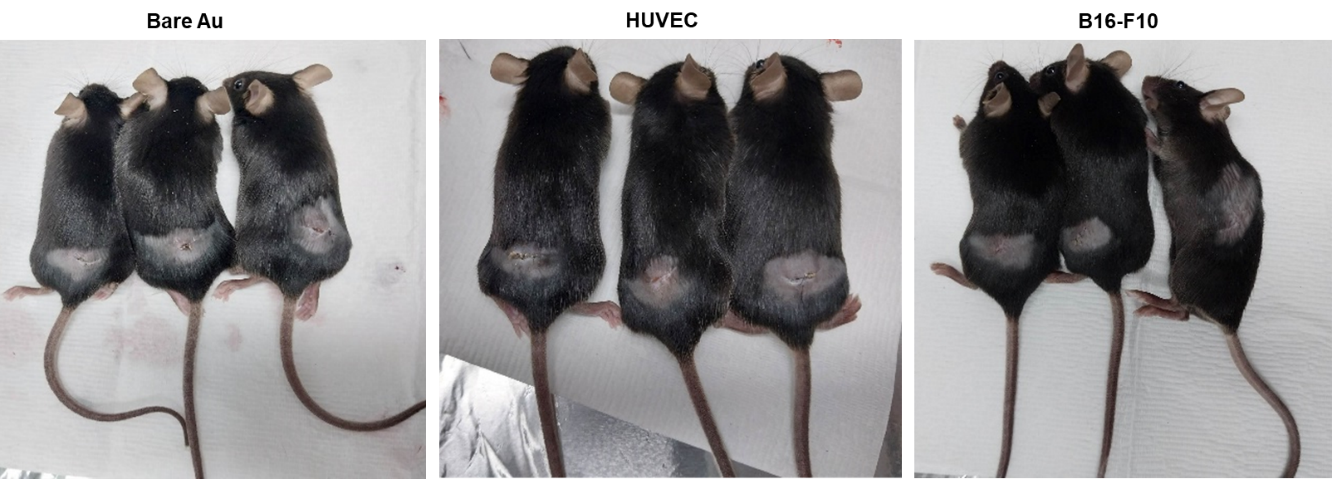


Fig. S9. Photos of implanted mice showing the healing of the surgical site without infection.

Table S1. Primers used for real-time polymerase chain reaction analysis.

| Genes | Strand (5’→3’) | |
| --- | --- | --- |
|  | FW | RV |
| mGAPDH | ATG TGT CCG TCG TGG ATC TGA | TGC CTG CTT CAC CAC CTT CT |
| miNOS | GGA GCG CTC TAG TGA AGC AA | TGC AGC TTG TCC AGG GAT TC |
| mIL1β | TGA GCT GGC CAG TGA AAT GA | AGA TTC GTA GCT GGA TGC CG |
| mTNFα | CAC AGT GAA GTG CTG GAC AC | GAT CAA AGC TGT AGG CCC CA |
| mCD 206 | ATG GAT TGC CCT GAA CAG CA | TGT ACC GAC CCC TCC ATC TA |
| mArg1 | GAT TAT CGG AGC GCC TTT CT | CCA CAC TGA CTC TTC CAT TCT T |
| mVEGF | CAG GAA TCC CAG AAA CAA CC | CAG GCT GCT CTA ACG ATG AA |
| mIL6 | GAGGATACCACTCCCAACAGACC | AAGTGCATCATCGTTGTTCATACA |
| mIL10 | ACTGGCATGAGGATCAGCAG | CTCCTTGATTTCTGGGCCAT |
| mCcl1 | GTTCTTGGCTCCACCAGACA | CATCCTGTATCCACACGGCA |
| hGAPDH | TGCACCACCAACTGCTTAGC | GCCATGGACTGTGGTCATGAG |
| h VWF | GCAGTGGAGAACAGTGGTG | GTGGCAGCGGGCAAAC |
| hBax | ATGTTTTCTGACGGCAACTTC- | AGTCCAATGTCCAGCCCAT |
| hBcl-2 | ATGTGTGTGGAGACCGTCAA | GCCGTACAGTTCCACAAAGG |
| hAKT | ACTCATTCCAGACCCACGA | AGCCGAAGTCCGTTATCT |
| hERK1/2 | GAACTCCAAAGGCTATACCAA | GAGGGCAGAGACTGTAGGTA |
| hPI3K | GAAGTTGCTCTACCCAGTGT | TGATAGCCGTTCTTTCATTTG |
| hVEGFR2 |  |  |
| hAng-1 | CAGAAAACAGTGGGAGAAGATATAACC | TGCCATCGTGTTCTGGAAGA |
|  |  |  |
